# Trigger finger – Poor outcome of surgery associated with younger age, pain, psoriatic arthritis and atopic disease

**DOI:** 10.48101/ujms.v129.10361

**Published:** 2024-09-12

**Authors:** Björn Holm, Johan Rönnelid, Eva Baecklund, Monica Wiig

**Affiliations:** a Department of Surgical Sciences, Uppsala University, Uppsala, Sweden; b Department of Immunology, Genetics and Pathology, Uppsala University, Uppsala, Sweden; c Department of Medical Sciences, Uppsala University, Uppsala, Sweden

**Keywords:** Trigger finger, outcome, atopy, diabetes, rheumatic disease

## Abstract

**Background:**

Trigger finger, or stenosing tendovaginitis, is one of the most common causes of hand disability, where a finger or thumb painfully snaps and locks due to a tendon-sheath size mismatch at the A1 pulley. The exact aetiology of trigger finger is unknown, though it is associated with factors like diabetes, rheumatic disease and carpal tunnel syndrome. The main purpose of this prospective study was to explore clinical characteristics and comorbidities in a cohort of 139 patients who underwent surgery for trigger finger and find factors of importance for the outcome 1 year postoperatively.

**Methods:**

Pain, range of motion, hand function evaluated by the Disabilities of the Arm Shoulder and Hand questionnaire as well as Quinnell grade of triggering were examined preoperatively. Symptom duration, working status, medical history and comorbidities at baseline were also noted. Further, range of motion was evaluated 3 months after surgery, pain and hand function were evaluated 3 and 12 months after surgery. An outcome scale with three levels was defined. The development of any new comorbidities was monitored during an extended postoperative observation period, with a mean duration of 70 months (range: 56–88 months).

**Results:**

Poor outcome was strongly associated with younger age (*P* = 0.0009), a high level of preoperative pain in the operated hand (*P* = 0.0027), psoriatic arthritis (*P* = 0.021) and atopic disease (*P* = 0.028; odds ratio [OR]: 3.87, 95% confidence interval [CI]: 1.15–13.04). A low range of motion preoperatively did not affect the outcome. Carpal tunnel syndrome was the most common comorbidity but did not affect the outcome. A good preoperative range of motion, good hand function and less pain were associated with better outcomes.

**Conclusion:**

Younger age, a high level of preoperative pain, psoriatic arthritis and atopic disease were factors associated with a worse outcome of trigger finger surgery. Pain and disability decreased 3 months postoperatively and continued to decrease between 3 and 12 months.

## Introduction

Trigger finger or stenosing tendovaginitis is one of the most common pathologies of the hand, characterised by an often painful snapping and locking of a finger or thumb. The biomechanical background is a disproportion between the diameter of the flexor tendons and the surrounding tendon sheath, typically occurring at the entrance of the sheath where it is reinforced by the first annular ligament, the A1 pulley.

Multiple risk factors have been suggested for trigger finger, including diabetes, rheumatic disease, carpal tunnel syndrome, repetitive strain of the hand, but many authors simply consider the condition as idiopathic (1–3).

The treatments comprise corticosteroid injection, splinting and surgical division of the A1 pulley. Surgery is the most effective method with reported success rates between 43 and 96% (4–6). The wide variety in results is partly dependent on differences in definitions of treatment success and follow-up duration but is also due to individual patient factors (7, 8).

Although trigger finger surgery is one of the most common procedures performed by hand surgeons, there are still only few studies focussing on factors that may affect the outcome of surgery. Knowledge of such factors could be a help to improve postoperative outcome.

To further expand the knowledge of factors of importance for outcomes of trigger finger surgery, the aim of this study was to explore detailed clinical characteristics and comorbidities of patients who underwent A1 pulley release, describe outcome at 3 and 12 months postoperatively and relate outcome to the baseline characteristics.

## Methods

In this prospective, single-centre study, patients older than 18 years of age who were operated for trigger finger at a university hospital were included. Exclusion criteria were concomitant Dupuytren’s contracture and steroid injection to the hand to be operated less than 1 month preoperatively. A hand surgeon examined patients referred to the clinic for trigger finger and those assessed to need surgery were invited to participate in the study. Written informed consent was obtained from all patients, and the study had approval of the regional research ethics board (approval number 2012/386).

Initially, 160 patients with trigger finger were invited and 139 were included in the study. The remaining 21 patients had declined participation (*n* = 8), improved spontaneously (*n* = 7) or had surgery postponed due to concurrent disease (*n* = 6).

Each patient completed three questionnaires at inclusion. The demographic questionnaire included dominant hand, heredity, occupation, working status, previous history of trigger finger, smoking, sick leave, comorbidities and concurrent treatments. Another questionnaire included self-assessment of the severity of symptoms in each finger including the degree of triggering adapted for Quinnell-grading with 1, normal movement; 2, uneven movement; 3, actively correctable snapping; 4, passively correctable locking and 5, fixed deformity (9), and pain by visual analogue scale (VAS: 0–9). The third was the DASH (Disabilities of the Arm Shoulder and Hand) questionnaire (scores 0–100) (10). The two first questionnaires were created for the purpose of this study and were complemented by patient interviews.

A certified hand therapist measured the range of motion individually in all finger joints, grip strength and pinch strength. A blood sample was collected at the time of surgery in order to analyse C-reactive protein (CRP), P-calprotectin, fructosamine and HbA1c to be used as tentative independent prognostic markers. Workload was categorised as shown in Figure 1a.

**Figure 1a:**
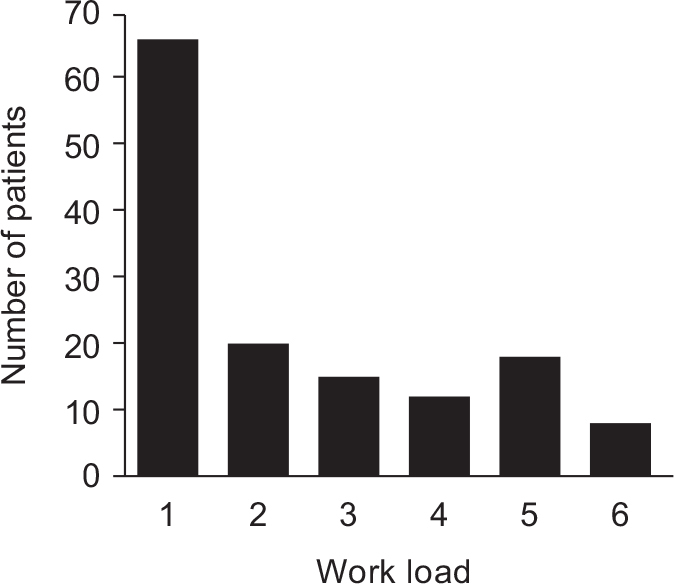
Self-reported workload among the 139 patients. 1. Retired/unemployed/ student, *n* = 66 (47%), 2. Office work, *n* = 20 (14%), 3. Office work plus some manual work, *n* = 16 (12%), 4. Light manual work, *n* = 11 (8%), 5. Heavy manual work, *n* = 18 (13%), 6. Very heavy manual work, *n* = 8 (6%).

**Figure 1b:**
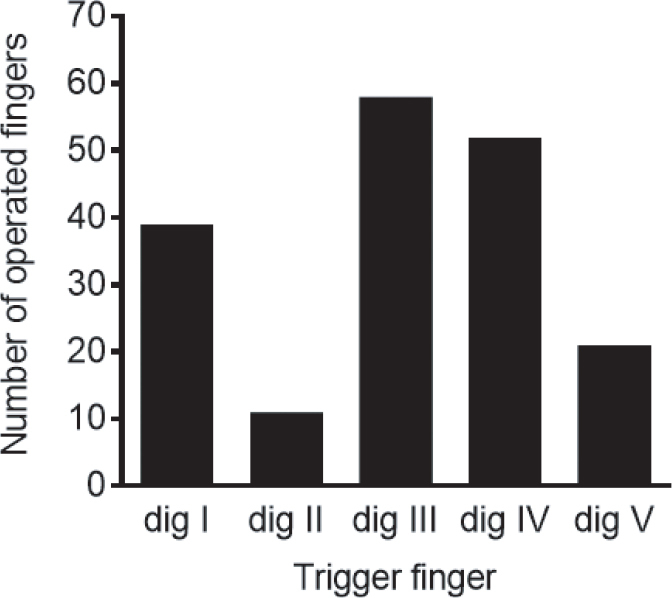
Distribution of operated fingers among the 139 patients (*n* = 181). Dig I 39, II 11, III 58, IV 52, V 21.

### Treatment

The operations were performed by two experienced specialists in hand surgery. In local infiltration anaesthesia and under tourniquet control, a transverse incision was made just distal to the distal palmar crease of the trigger finger. As for the trigger thumbs, an angled incision was made on the flexor crease of the metacarpophalangeal joint. The A1 pulley was exposed by blunt scissor dissection and released by a longitudinal incision. The release was checked by the patient moving the finger to ensure there was no remaining triggering. The skin was closed with single stitches using a 5.0 non-absorbable monofilament suture, and a dressing was applied. Early active motion was encouraged. No splint was used postoperatively.

### Follow-up

Three months after surgery all patients were seen by the surgeon and the hand therapist. The questionnaires regarding pain, Quinnell-grade and DASH, and measurements of range of motion (ROM) and strength were repeated. Any complications were noted.

One year post surgery the self-assessment and DASH questionnaires were sent to the patients. With two missing assessments for Quinnell and pain and four missing for DASH, the 1 year follow up rate was 99% for Quinnell and pain and 97% for DASH.

Patient files were observed for any development of new comorbidities until February 2021. The mean total postoperative observation period was 70 months (range: 56–88). The study outline is presented in Table 1.

**Table 1:** Outline of study design.

Assessment	Baseline/ pre-op	Three months follow-up	One year follow-up	End of observation period
Patient questionnaire	x			
Blood sample	x			
Range of motion	x	x		
Grip and pinch strength	x	x		
Quinnell grade	x	x	x	
Pain by VAS	x	x	x	
DASH	x	x	x	
Outcome-scale			x	
Clinical data from patient records	x			x

VAS: Visual Analogue Scale, patients’ self-assessment of pain; DASH: Disabilities of the Arm, Shoulder and Hand-questionnaire.

### Outcome scale

To get a composite measure of the main outcome measures used in clinical practice, we constructed an outcome scale consisting of pain, ROM and triggering by Quinnell grade. Three levels of outcome were defined: excellent, fair and poor.

Excellent outcome was defined as no triggering and no pain in the operated finger(s) 1 year after surgery, and a ROM greater or equal to the median ROM of the corresponding unaffected control fingers at 3 months.

Poor outcome was defined as any triggering or pain in the operated finger 1 year after surgery, regardless of ROM.

The intermediate group with no triggering and no pain after 1 year, but a ROM less than the corresponding control fingers at 3 months were defined as having fair outcome.

### Statistics

For univariate analyses, we uniformly used non-parametric statistics, and data were expressed as medians with inter quartile ranges (IQRs) or minimum to maximum range. For clarity, descriptive information was also shown as mean values with standard deviations. The Mann-Whitney U test was used to compare quantitative baseline characteristics by excellent versus non-excellent, and poor versus non-poor outcome. Dichotomous clinical characteristics were evaluated with Chi2 or Fisher’s exact test when appropriate. Parallel multivariate investigations with excellent versus non-excellent outcome, and poor versus non-poor outcome as dependent variables respectively, were also performed with logistic regression expressed as OR with 95% CI, adjusting for age and sex. The paired Wilcoxon signed-rank test was used to evaluate changes in quantitative clinical variables over time. *P*-values < 0.05 were considered significant. No corrections for mass significance were made.

## Results

### Baseline characteristics

**Figure 2:**
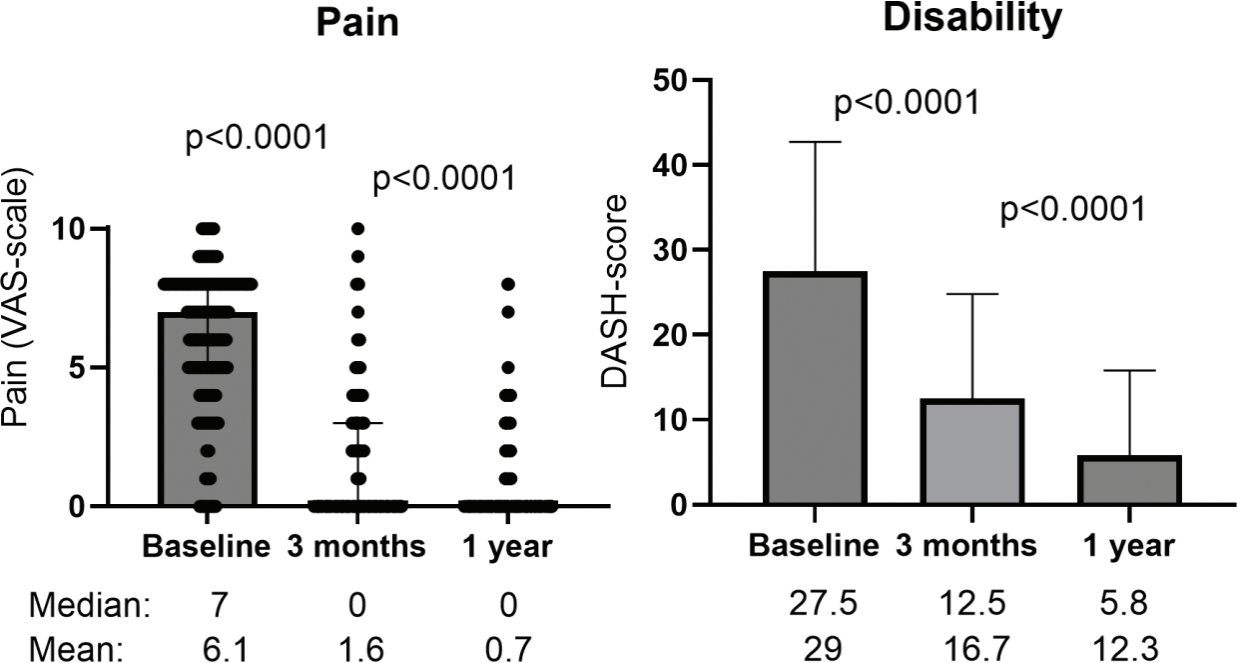
Box plots of pain by visual analogue scale (VAS), and disability by the DASH-questionnaire (Disability of the Arm Shoulder and Hand) measured at baseline, 3 months and 1 year after surgery. Dispersion is shown as IQR.

**Figure 3:**
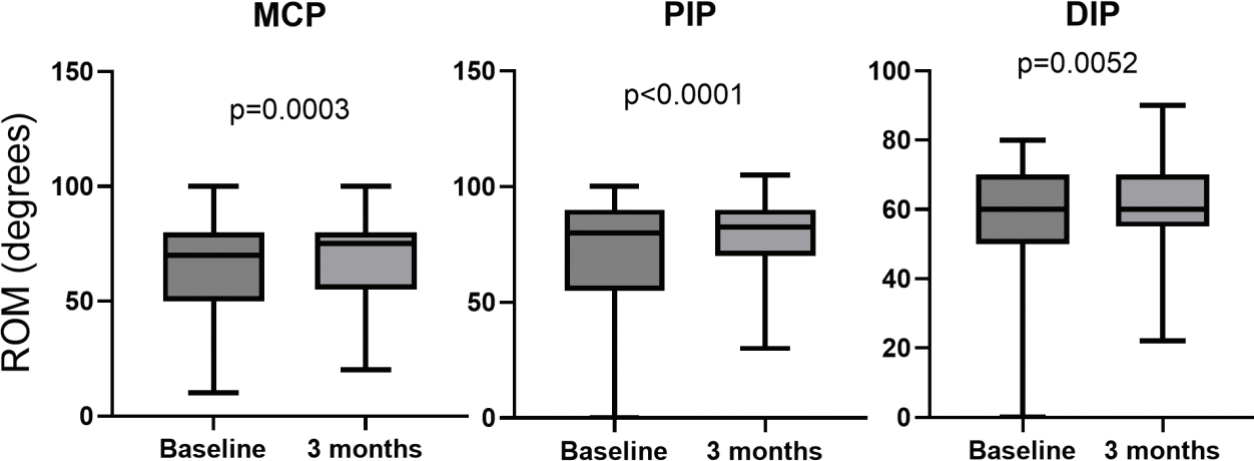
Box plots of range of motion (ROM) at baseline and 3 months post-surgery for the metacarpophalangeal (MCP), proximal interphalangeal (PIP) and distal interphalangeal (PIP) joints of the finger which was worst affected at baseline for each patient. Dispersion is shown as min to max.

The majority of the 139 included patients were women, the mean age at enrolment was 62 years, and 91% were right-handed. Clinical baseline characteristics and measurements are summarised in Table 2 and Figures 2 and 3.

**Table 2:** Baseline characteristics of the 139 patients operated for trigger finger.

Characteristics	Value
Women, *n* (%)	83 (60%)
Age, years (median [IQR]/mean [SD]/ range)	63 (56–69)/61.6 (10.8)/ 25–84
BMI (median [IQR]/mean [SD]). Data from 138 patients.	27.6 (25.2–30.5)/ 28.4 (4.9)
Dominant hand, *n* (%) (right/left/ ambidextrous)	126 (90.6%)/12 (8.6%)/1 (0.7 %)
Dominant hand operated, *n* (%) (*n* = 138*)	84 (61%)
First-degree relative with TF, *n* (%), Yes/no/not known	23 (17%)/57 (41%)/58 (42%)
Patients with previous trigger finger, *n* (%)	40 (29%)
Symptom duration, months (median [IQR]/ mean [SD])	10 (7–18)/ 16.7 (19.9)
Smoking status, *n* (%) (current/ex/never)	13 (9%)/61 (44%)/65 (47%)
Frayed tendon of the TF at surgery, number (%)	15 (11%)
Diabetes mellitus, *n* (%)	49 (35%)
Rheumatoid arthritis, *n* (%)	1 (1%)
Carpal tunnel syndrome, *n* (%) (history of/currently/ operated at the same time)	59 (42%)/20 (14%)/6 (4%)
Diabetes, RA or CTS, n (%)	86 (62%)
Atopy (asthma or eczema), *n* (%)	14 (10%)
CRP (median [IQR]/ mean [SD])	1.27 (0.58–3.24)/ 2.8 (4.3)

IQR: interquartile range; SD: standard deviation; TF: trigger finger; RA: rheumatoid Arthritis; CTS: carpal tunnel syndrome.

* One patient was ambidextrous and therefore excluded from the presentation of dominant hand.

None of the patients were on sick leave due to trigger finger at baseline. After surgery, 53 (87%) of the able-bodied patients younger than 65 years (*n* = 61) needed sick leave (median 24 days, range 2–159). Sick leave was significantly shorter among men (median 21 days) than among women (median 24 days) (*P* = 0.038). It was longer among patients with heavy or very heavy working tasks (median 30 days) compared to those with lighter working tasks (median 21 days) (*P* < 0.0001).

Young age was associated with more preoperative pain, when comparing the younger with older patients split at the median age of 63 years (median VAS 8 and 6 respectively; *P* = 0.0003). Twenty-six of the patients (19%) had multiple fingers operated and the total number of operated fingers was 181. The middle finger was most commonly affected (Figure 1b).

The median preoperative trigger finger symptom duration was 10 months (range: 1–124 months). Fifteen patients (11%) had a frayed superficial trigger finger flexor tendon which associated with long symptom duration (median 23 months as compared to 9 months for the remaining patients; *P* = 0.0003). A frayed tendon was associated with less improvement of pain at 1 year after surgery (*P* = 0.04), but did not affect the overall outcome.

At baseline, ROM was smaller in all joints of the affected fingers compared to the median of the corresponding unaffected control-fingers (*P* < 0.0001). Comorbidities were common. Only 5 (4%) of the patients had no reported comorbidity or drug treatment at baseline. Sixty-two percent of the patients had at least one of the three diagnoses previously known to be associated with trigger finger: diabetes, rheumatoid arthritis or a history of carpal tunnel syndrome.

Asthma and eczema were present in 11 and 6% of the patients, respectively. Asthma and eczema were, after careful chart review, further divided into atopic and non-atopic disease. Twelve patients (9%) had atopic asthma and three (2%) had atopic eczema, in total 14 (10%) had any of these atopic diseases at baseline.

### Follow-up

Stitches were removed 2–3 weeks postoperatively without any infections or wound healing problems needing treatment. Eighteen patients reported some numbness in an operated finger at this stage. None of the patients had any remaining triggering.

After 3 months, there was a significant decrease in pain and disability measured by DASH (Figure 2). Two patients had some reappearing triggering in the operated finger.

Range of motion was significantly increased in all joints of the operated fingers, although numerical changes were small. Patients with a low preoperative ROM, had the greatest improvement (Figure 3). Interestingly, the sum of ROM in metacarpophalangeal (MCP), proximal interphalangeal (PIP) and distal interphalangeal joint (DIP) also increased marginally in the contralateral asymptomatic control-fingers postoperatively (*P* = 0.03).

There was a significant increase of pinch-strength but not of grip-strength after 3 months when all patients were evaluated together. Split by operated finger, there were highly significant increases in pinch-strength for the patients with operated thumbs and middle fingers (Table 3).

**Table 3:** Pinch- and grip-strength.

Operated finger	Baseline	After 3 months	*P*
**Pinch-strength (kg)**			
Any *n* = 138	6.8 (4.8–8.4)	7.0 (5.7–9.0)	**< 0.0001**
I *n* = 32	4.9 (3.7–7.7)	6.7 (5.2–9.1)	**< 0.0001**
II *n* = 3	9.5 (5–11.5)	9.2 (4.0–11.5)	0.50
III *n* = 37	6.5 (5.1–8.0)	7.2 (5.8–8.5)	**0.0008**
IV *n* = 30	7.2 (5.8–8.1)	7.0 (5.8–8.0)	0.40
V *n* = 9	7.2 (1.9–8.9)	7.3 (4.9–9.7)	0.36
**Grip-strength (kg)**			
Any *n* = 138	23.0 (13.3–31.7)	21.9 (16.7–30)	0.76
I *n* = 32	26.0 (14.7–33.5)	27.7 (19.2–36.5)	0.06
II *n* = 3	32.0 (12.0–40.3)	30.0 (13.3–30.7)	0.75
III *n* = 37	20.0 (11.0–30.7)	20.0 (14.1–28.0)	0.92
IV *n* = 30	21.7 (13.3–28.4)	20.0 (14.0–27.0)	0.55
V *n* = 9	21.3 (6.9–37.3)	29.3 (12.7–35.9)	0.82

Quantitative data are shown as median (IQR). Significant *P*-values are depicted in bold. Patients with more than one operated finger are excluded from calculations for individual fingers (*n* = 26) as well as one patient missing the 3 months-data.

Pain and DASH had further improved at 1 year post-operatively (Figure 2). Seven patients (5%) had a relapse of triggering in an operated finger. Using the constructed outcome scale, the outcome was excellent in 30 (22%), fair in 70 (50%) and poor in 39 (28%) of the patients.

During the extended postoperative observation period, 51 patients (37%) had either development of a new trigger finger or a relapse of triggering in the operated finger. One patient developed diabetes. Four patients developed a rheumatic disease (two rheumatoid arthritis (RA), one systemic lupus erythematosus (SLE), and one psoriatic arthritis (PsoA)). Fifteen patients (11%) developed a new carpal tunnel syndrome. Further, 4 patients were diagnosed with atopic asthma and 2 with atopic eczema, whereof 2 were diagnosed with both diseases and 1 already had a previous atopic diagnosis. In effect, 3 more patients were diagnosed with atopy during the observation period.

### Factors associated with outcome of surgery

In univariate analyses, poor outcome, as compared to non-poor outcome, was associated with younger age, more pain in the operated finger, and PsoA or atopic disease (atopic asthma and/or atopic eczema) at baseline. When including patients who were diagnosed with PsoA or atopic disease, respectively during the postoperative observation period, both associations became stronger. In multivariate logistic regression analyses, adjusted for age and sex, these associations remained (Tables 4 and 5).

**Table 4:** Outcome of trigger finger surgery for patients with different clinical characteristics.

Characteristics at baseline/before surgery	Excellent outcome	Fair outcome	Poor outcome	*P*, OR (CI) Excellent outcome vs. non-excellent (univariate analyses)	*P*, OR (CI) Poor vs. non-poor outcome (univariate analyses)	*P*, OR (CI) Excellent outcome vs. non-excellent (multivariate analyses)	*P*, OR (CI) Poor vs. non-poor outcome (multivariate analyses)
Number of patients (total *n* = 139)	30	70	39	N/A	N/A	N/A	N/A
Women, *n* (% in each outcome group)	21 (70%)	40 (57%)	22 (56%)	0.19 0.56 (0.24–1.35)	0.62 1.21 (0.57–2.56)	N/A	N/A
Age (median/mean)	65/63.2	64.5/63.7	56/56.7	0.28	**0.0009**	N/A	N/A
Previous trigger finger, *n* (%)	11 (37%)	22 (31%)	7 (18%)	0.28 1.60 (0.68–3.76)	0.08 0.44 (0.18–1.11)	0.26 1.66 (0.69–3.97)	0.11 0.47 (0.18–1.22)
BMI (median/mean). Data from 138 patients.	27.0/27.3	27.8/28.3	28.0/29.4	0.27	0.19	0.18	0.27
High workload (heavy or very heavy working tasks), *n* (%)	5 (17%)	11 (16%)	10 (26%)	0.75 0.84 (0.29–2.45)	0.19 1.81 (0.74–4.43)	0.90 1.08 (0.32–3.70)	0.70 0.81 (0.28–2.35)
Symptom duration months (median/mean)	12/22	9/14	13/17	0.34	0.17	0.10	0.73
Frayed tendon in main operated finger, *n* (%)	3 (10%)	6 (9%)	6 (15%)	0.87 0.90 (0.24–3.41)	0.28 1.84 (0.61–5.56)	0.87 0.90 (0.23–3.51)	0.16 2.42 (0.73–8.00)
DASH score (median/mean)	15/21.1	29.7/30.8	35/32.2	**0.0021**	0.21	**0.0023**	0.27
Quinnell grade (median/mean)	4/3.9	4/3.9	4/3.9	0.91	0.94	0.83	0.70
Pain VAS main operated finger (0–1) (median/mean)	5/5.0	7/6.1	8/6.9	**0.020**	**0.024**	**0.018**	0.083
Pain VAS (sum of all 10 fingers) (median/mean)	6/5.9	8/7.6	8/10.5	**0.0075**	**0.0149**	**0.0081**	**0.0027**
ROM MCP (median/mean)	80°/72°	65°/62°	65°/63°	**0.0023**	0.42	**0.0035**	0.73
ROM PIP (median/mean)	85°/76°	70°/64°	80°/72°	**0.0317**	0.54	0.08	0.33
ROM DIP (median/mean)	65°/56°	55°/55°	55°/56°	0.21	0.58	0.80	0.73
Grip strength operated hand, kg (median/mean)	20/22.2	23.15/23.6	23.7/26.3	0.49	0.63	0.85	0.96
Pinch strength operated hand, kg (median/mean)	6.5/6.1	6.75/6.6	7/6.9	0.38	0.55	0.95	0.96
CRP (median/mean)	1.2/1.7	1.4/2.9	1.1/3.5	0.14	0.54	**0.033**	0.33
Calprotectin (median/mean)	0.46/0.61	0.53/0.60	0.57/0.65	0.27	0.35	0.70	0.58
Diabetes, *n* (%), *n* = 49	6 (12%)	29 (59%)	14 (29%)	**0.048** 0.38 (0.14–1.02)	0.92 1.04 (0.48–2.25)	0.055 0.40 (0.15–1.07)	0.81 0.91 (0.40–2.04)
HbA1c (median/mean)	38/41.3	40.5/45.2	40/48.7	0.06	0.16	0.060	0.18
Fructosamine (median/mean)	286/296	301/309	291/322	0.15	0.87	0.12	0.37
RA at baseline, *n* (%)	0	1 (1%)	0	NA	NA	NA	NA
RA at baseline or observation period, *n* (%)	0	1 (1%)	2 (5%)	1.0*	0.19*	0.20**	0.094 8.04 (0.63–102)
PsoA at baseline, *n* (%)	0	0	3 (8%)	1.0*	0.021*	0.26**	0.021**
PsoA at baseline or observation period, *n* (%)	0	0	4 (10%)	0.58*	0.0055*	0.17**	0.0035**
History of carpal tunnel syndrome, *n* (%), *n* = 59	10 (17%)	31 (53%)	18 (30%)	0.25 0.61 (0.26–1.43)	0.58 1.23 (0.59–2.60)	0.22 0.58 (0.24–1.40)	0.99 1.00 (0.45–2.24)
Osteoarthritis in hands at baseline, *n* (%)	4 (13%)	8 (11%)	8 (20%)	0.85 0.89 (0.28–2.91)	0.20 1.89 (0.71–5.06)	0.60 0.73 (0.22–2.44)	0.085 2.61 (0.89–7.67)
Osteoarthritis except hands, *n* (%)	8 (27%)	18 (26%)	10 (26%)	0.91 1.05 (0.42–2.63)	0.97 0.98 (0.42–2.29)	0.85 0.91 (0.35–2.36)	0.50 1.37 (0.55–3.37)
Gout & pseudogout, *n* (%)	1 (3%)	3 (4%)	1 (3%)	1.00*	1.00*	0.93 0.91 (0.09–8.77)	0.95 0.93 (0.10–9.04)
Chronic pain*** *n* (%)	7 (23%)	18 (26%)	13 (33%)	0.58 0.77 (0.30–1.97)	0.32 1.50 (0.67–3.36)	0.55 0.75 (0.29–1.96)	0.42 1.42 (0.61–3.29)
Depression ever, *n* (%)	5 (17%)	17% (12)	15% (6)	0.98 1.01 (0.34–2.99)	0.82 0.89 (0.32–2.45)	0.76 1.19 (0.39–3.69)	0.36 0.61 (0.21–1.78)
Hypothyroidism or supplementation ever, *n* (%)	2 (7%)	13 (19%)	5 (13%)	0.17 0.36 (0.08–1.65)	0.74 0.83 (0.28–2.47)	0.13 0.33 (0.07–1.60)	0.17 0.43 (0.12–1.51)
Hyperlipidemia or statin use, *n* (%)	9 (30%)	28 (40%)	14 (36%)	0.39 0.68 (0.29–1.63)	0.90 0.95 (0.44–2.06)	0.33 0.65 (0.27–1.58)	0.84 1.09 (0.49–2.44)

VAS: visual analogue scale; OR: odds ratio; CI: confidence interval; BMI: body mass index; ROM: Range of Motion; MCP: metacarpophalangeal joint; PIP: proximal interphalangeal joint; DIP: distal interphalangeal joint; DASH: Disabilities of the Arm Shoulder and Hand; CRP: C-reactive protein; RA: rheumatoid arthritis; PsoA: psoriatic arthritis.

Significant *P*-values are depicted in bold.

* Fisher’s exact test.

** OR and CI could not be calculated by the statistics’ software since there was zero patients in one group.

*** Including tendinitis, enthesitis, epicondylitis, fibromyalgia, muscle pain, neuropathic pain, lumbago.

Univariate analyses were performed with the Mann-Whitney’s U test for quantitative characteristics and with Chi2 test or Fisher’s test for qualitative characteristics. Multivariate analyses were performed with logistic regression, with adjustments for age and sex, and results are given both as *P*-values and odds ratios (OR) with 95% confidence intervals (CI) for nominal data. Quantitative variables are given as median (inter quartile range)/mean (standard deviation).

**Table 5:** Outcome of trigger finger surgery for patients with atopy.

Atopic disease	Excellent outcome	Fair outcome	Poor outcome	*P*, Excellent outcome vs. non-excellent	*P*, Poor vs. non-poor outcome	*P*, OR (CI) Excellent outcome vs. non-excellent (multivariate analyses)	*P*, OR (CI) Poor vs. non-poor outcome (multivariate analyses)
Atopic asthma at baseline, *n* (%)	0	6 (9%)	6 (15%)	0.06	0.08	0.0100**	0.0958 2.97 (0.83–10.58)
Atopic asthma at baseline or observation period, *n* (%)	0	7 (10%)	9 (23%)	**0.0257**	**0.0076**	0.0027**	**0.0106** **4.35 (1.40–13.56)**
Atopic eczema at baseline, *n* (%)	0	1 (1%)	2 (5%)	1.00*	0.19*	0.19**	0.18 5.74 (0.40–82.28)
Atopic eczema at baseline or observation period, *n* (%)	0	1 (1%)	4 (10%)	0.58*	0.0219*	0.11**	**0.0176** **11.88 (1.15–122.4)**
Atopy (asthma or eczema) at baseline, *n* (%)	0	6 (9%)	8 (21%)	**0.0385**	**0.0106**	0.0050**	**0.0279** **3.87 (1.15–13.04)**
Atopy at baseline or observation period, *n* (%)	0	7 (10%)	10 (26%)	**0.0210**	**0.0026**	0.0019**	**0.0070** **4.60 (1.50–14.16)**

OR: odds ratio; CI: confidence interval.

Significant *P*-values are depicted in bold.

* Fisher’s exact test.

** OR and CI could not be calculated by the statistics’ software since there was zero patients in one group.

Multivariate analyses were performed with logistic regression, with adjustments for age and sex, and results are given both as *P*-values and odds ratios (OR) with 95% confidence intervals (CI).

Baseline factors associated with an excellent outcome, as compared to non-excellent outcome, were a better DASH score, less pain, a wider ROM in the operated finger and a low CRP. Among the patients with an excellent outcome, there were fewer patients with asthma and atopy than among those with non-excellent outcome. Patients with diabetes were less likely to have an excellent outcome compared to non-excellent in univariate analysis, but this association disappeared after adjustment for age and sex, with a *P*-value close to the level of significance (*P* = 0.055) (Tables 4 and 5).

The level of pain and the lack or presence of atopic disease were the strongest predictors for both an excellent and poor result. Moreover, all associations to atopy (atopic asthma, atopic eczema or any atopic disease) increased when patients developing atopy during the follow-up period were also included (Table 5).

Further, we found no significant associations between outcomes and reported trigger finger in first-degree relatives, ever smoking, number of steroid injections injected more than 1 month and less than 6 months preoperatively and psychiatric disorders other than depression (data not shown).

## Discussion

The worst outcome, defined as poor in the outcome scale, was associated with younger age, more pain in the operated finger at baseline, and the presence of psoriatic arthritis or atopic disease.

An association between younger age and poor outcome could suggest a more severe and difficult-to-treat trigger finger disease in younger patients, which is further supported by the finding of more pain in the younger patients. Also suggestive of a link between age and outcome of surgery, Koopman et al. reported an association between older age and less pain 3 months after trigger finger surgery, and likewise van den Berg et al. found an association between older age and higher success rate after steroid injection for trigger finger (11, 12).

Baseline pain was the best predictor of a poor outcome of surgery when analysed for all patients together, a finding in line with other studies (13).

An association between PsoA and poor outcome of trigger finger surgery has to our knowledge not been reported previously but is consistent with well-described features of tendinopathy in PsoA, such as tenosynovitis and dactylitis. Recent ultrasound studies have also identified thickening of A1 pulleys in patients with PsoA (14) and in PsoA-related dactylitis (15), and it is plausible that continuous immune activity may result in recurrent tenosynovitis symptoms in operated PsoA patients.

Rheumatoid arthritis has previously proven to be associated with worse outcome after trigger finger surgery (12). As in PsoA, a continued disease-linked tendency to develop tenosynovitis has been suggested to disturb outcomes of surgery. Only one patient had RA at baseline in our study, precluding further assessments of RA and outcome.

A recent study reported that psychological variables like anxiety and depression appeared to predict disability by DASH after conservative trigger finger treatment (16). In our study of surgical treatment, we could not detect corresponding findings.

An association between atopic disease and outcome of trigger finger surgery has, like PsoA, to our knowledge not been reported previously. Atopy is a genetically imposed tendency of developing asthma, eczema and other manifestations of allergy. These diseases are characterised by immune dysregulation and low-grade inflammation, and the disturbed immunologic pathways have been described to be shared also with other inflammatory and autoimmune diseases (17). Recent research has revealed that systemic inflammation associated with allergic conditions can compromise tendon structure and function. Analysing data from a large-scale health survey, the researchers found a correlation between allergies and the risk of developing tendinopathy (18). Our findings further substantiate the link between atopy and detrimental effects on tendon function. In addition, lack of atopy was the best predictor of an excellent outcome. As atopic diseases are common in the population, our results may apply to a considerable number of trigger finger patients.

In the best outcome group, there was a smaller proportion of diabetics compared to the group with non-excellent outcome. This suggests that diabetes may have a negative impact on outcome, but the effect seems limited as the significance disappeared in multivariate analyses, adjusted for age and sex. Further, we did not find an association between poor outcome and diabetes, nor did we find an association between the severity of diabetes measured as level of HbA1c and outcome. Ho et al. arrived at similar conclusions (19). In that study, comparing clinical outcomes of trigger finger surgery in patients with and without diabetes, there was a low and similar complication rate and a high rate of postoperative satisfaction in both groups.

A wide range of motion predicted excellent outcome, suggesting a less severe trigger finger disease in patients with less stiffness. This finding was particularly robust for the MCP-joints. However, a low ROM at baseline did not predict poor outcome. Thus, the treatment yields a greater improvement for the patients with the stiffest fingers, giving these patients good hope of recovery.

In multivariate analyses, there was a significant association between a lower level of CRP and an excellent outcome. Since CRP is an indicator of inflammation, this suggests an association between inflammation and worse outcome.

Consistent with the findings of Baek et al., we found that the presence of a frayed superficial flexor tendon at surgery was correlated to less improvement in postoperative pain (20). These patients also had a significantly longer duration of symptoms prior to surgery. In general, however, we did not detect an association between preoperative symptom duration and outcome of surgery.

Contrary to the findings of Koopman et al., who identified preoperative steroid injections as one of the most influential factors for poorer outcomes in terms of pain and hand function after surgery (12, 21), we did not find any such association in our study.

The strengths of this study include its detailed, prospective design with clinical characteristics from patient questionnaires supplemented by a structured patient interview and medical chart review, the long and consistent follow-up at one centre, and almost no missing data. The composite outcome scale aimed to represent the clinical scenario accurately, integrating the common evaluation variables triggering, pain and ROM. It can also be noted that the long follow-up of 1 year revealed a continued improvement in DASH-score and pain also beyond the 3-month follow up. Additionally, the detailed clinical data collection highlighted a notable burden of comorbidity, including atopy, among the study participants. Atopy has previously not been explored in the context of trigger finger.

Limitations of the study include the relatively small number of included patients and that some of the baseline factors were present in only a few patients and therefore could not be evaluated. The self-grading questionnaire created for the study is not validated, but is based on the Quinnell-grading, which has been used to validate other scales relevant for trigger finger (22, 23). Range of motion, grip and pinch strength were only measured at inclusion and at 3 months. Thus, we cannot exclude that ROM has changed after the 3-month assessment. However, as ROM is not included in the definition of poor outcome this uncertainty does not affect the main results of the study. The rather low number of patients included in the multivariate evaluations, where each independent variable individually had been adjusted for age and sex, is also a limitation.

In conclusion, this study identified factors predicting worse outcome after trigger finger surgery, including younger age, increased preoperative pain, and the presence of atopic disease. These findings underscore the importance of providing special attention to such patients both before and after surgery to optimise treatment outcomes.
